# The Relationship Between Healthcare System Distrust and Intention to Use Violence Against Health Professionals: The Mediating Role of Health News Perceptions

**DOI:** 10.1111/hex.70151

**Published:** 2025-01-10

**Authors:** Selman Kızılkaya, Büşra Buğdali

**Affiliations:** ^1^ Department of Health Management Faculty of Economics and Administrative Sciences Dicle University Diyarbakır Türkiye; ^2^ Department of Health Management Dicle University Diyarbakır Türkiye

**Keywords:** health news, healthcare system distrust, violence

## Abstract

**Background:**

Health news refers to media coverage that informs the public about health‐related issues, policies and healthcare systems, shaping public perception and understanding. While prior research has examined media's impact on public health behaviour, limited studies have focused on how perceptions of health news affect attitudes towards healthcare professionals, especially in the context of violence against them. This study addresses this gap, examining the mediating role perception of health news on the relationship between distrust in healthcare systems and intentions to use violence against healthcare professionals.

**Aim:**

This research aims to explore how the perception of health news influences the relationship between distrust in healthcare systems and the intention to use violence against healthcare professionals.

**Methodology:**

A survey was conducted with 693 participants over the age of 18 who had received healthcare services in the last year. The study utilized an intermediary model to assess the role of perception of health news in the relationship between distrust in the healthcare system and the intention to use violence against healthcare professionals.

**Results:**

The findings indicate a positive correlation between distrust in healthcare systems and the intention to use violence against healthcare professionals. Additionally, the perception of health news was found to significantly mediate this relationship.

**Conclusion:**

The study concludes that negative perceptions of healthcare systems, exacerbated by the portrayal of health news, can escalate the risk of violence against healthcare professionals.

**Patient or Public Contribution:**

There was no direct patient or public involvement in the design, conduct, reporting, or dissemination plans of this research. The study primarily relied on data collected through surveys and questionnaires administered to participants. Although the research addresses issues pertinent to the public and healthcare professionals, such as violence against healthcare professionals and the role of media in shaping public perceptions, the public's role was limited to responding to the survey. The findings and implications of this research are intended to benefit the public and healthcare community by informing future strategies and interventions, but the public did not actively contribute to the research process itself.

## 1 Introduction

Distrust in healthcare systems has been increasingly observed across various countries, often attributed to perceived deficiencies in healthcare quality, accessibility challenges and unsatisfactory patient experiences [1]. Distrust in healthcare systems can erode societal trust in healthcare, leading to broader repercussions that undermine the stability and efficacy of public health systems [2]. Additionally, this mistrust can significantly undermine the effectiveness of healthcare services, as it impacts individuals' willingness to engage with healthcare providers, potentially resulting in delays in care and poorer health outcomes [3].

One of the severe consequences of distrust in healthcare systems is the growing trend of violence against healthcare professionals. In Turkey, as in many countries, healthcare professionals face elevated risks of workplace violence, often linked to dissatisfaction with service provision and unmet expectations [4]. Recent data underscore the critical nature of this issue, revealing that healthcare professionals face substantial risks of workplace violence globally, and this challenge is particularly severe in Turkey [1, 4, 5]. Notably, during the COVID‐19 pandemic, healthcare professionals, working on the frontline with direct interactions with patients and their companions, were exposed to various forms of violence [6, 7, 8]. Findings from recent studies indicate that while the incidence of workplace violence against healthcare professionals was relatively high during this period, it was lower compared to levels before the pandemic [9, 10]. Research by [5] indicates that healthcare workers are increasingly viewed as representatives of perceived failures within the healthcare system, which subjects them to heightened aggression from frustrated patients and their families. Additional studies highlight that violence towards healthcare providers is not only a widespread phenomenon but also one that manifests acutely within specific national contexts [3, 11].

The issue of violence against healthcare professionals is a pressing global concern that threatens both the integrity of healthcare systems and their safety. Addressing this issue requires comprehensive efforts to restore trust in healthcare systems and protect healthcare professionals from the adverse outcomes associated with public discontent and distrust [12]. At this point, the role of health news is of great importance. Health news is a critical tool for the public to learn about healthcare systems and healthcare professionals. Health news, which often dramatizes or sensationalizes healthcare‐related issues, contributes to an erosion of public trust, increasing the likelihood of aggressive attitudes towards healthcare professionals [13, 14]. Health news also holds a dual role in shaping public perceptions, acting both as a source of information and as a powerful tool that influences societal attitudes. Research consistently shows that repeated exposure to negative narratives in the health news can exacerbate distrust in healthcare systems, creating a climate where hostile behaviours towards healthcare providers become normalized [15, 16].

However, while media has the potential to deepen distrust and amplify aggressive tendencies, responsible health news could serve as a counterbalance by fostering trust and offering accurate, balanced narratives. Health news can mitigate public fear and aggression, underscoring the importance of accurate information in shaping constructive public attitudes towards healthcare systems [17, 18]. Health news can be an agent for trust‐building, sensationalized coverage of healthcare issues may foster a sense of distrust and disappointment, particularly in settings where healthcare systems face public scrutiny [19, 20].

While previous research has extensively documented the general trends in healthcare‐related violence and the role of distrust in healthcare systems, there is a notable gap regarding how the perception of health news specifically influences distrust in healthcare systems and, in turn, the likelihood of violence against healthcare professionals. This study addresses this critical gap by examining how the perception of health news influences the relationship between distrust in healthcare systems and the intention to use violence against healthcare professionals. Focusing on Turkey provides a unique opportunity to explore how local sociocultural dynamics intersect with global trends, offering insights that may inform both national and international policy strategies aimed at protecting healthcare workers and enhancing trust in healthcare systems. This research thus contributes to an emerging body of evidence on the perception of health news' impact on the use of violence against healthcare professionals and distrust in healthcare systems [21, 22].

### 1.1 Aim

This research aims to explore how the perception of health news influences the relationship between distrust in healthcare systems and the intention to use violence against healthcare professionals.

## 2 Methodology

This study employs a cross‐sectional, descriptive and relational design to examine the mediating role of perception of health news in the relationship between distrust in healthcare systems and the intention to use violence against healthcare professionals.

### 2.1 Research Model and Hypotheses

**Figure 1:**
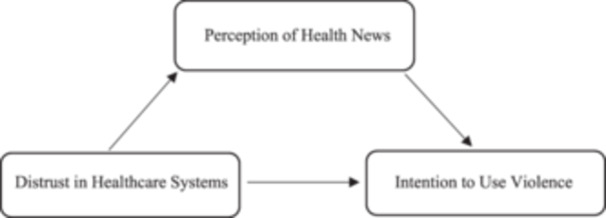
Research model.

In this study, the mediation model was used. This model expresses the mechanism through which the relationship between the predictor and the outcome variables occurs, rather than the ‘effect of a predictor variable on the outcome variable’ that was tried to be understood in the past [23, 24]. Accordingly, the study examines how the relationship between distrust in healthcare systems as the predictor variable and the intention to use violence against healthcare professionals as the outcome variable is shaped according to the perception of health news as the mediator variable. The model created based on this is presented as follows (Figure 1).

The hypotheses for this study were as follows:

Distrust in healthcare systems positively affects the intention to use violence against healthcare professionals.

The perception of health news negatively affects the intention to use violence against healthcare professionals.

The perception of health news mediates the relationship between distrust in healthcare systems and the intention to use violence against healthcare professionals.

### 2.2 Study Participants

Participants in this study were recruited through convenience sampling to reach a broad and accessible sample of individuals. Eligibility criteria required participants to be over the age of 18 and to have previously received healthcare services from any healthcare institution in Turkey. Each participant received comprehensive information about the study, including its purpose, objectives and the voluntary nature of participation, thereby ensuring informed consent. The study group of the research is 693 individuals over the age of 18 who received healthcare services from any healthcare institution in Turkey in the last year. The majority of respondents are male (61.6%) and have an average age of 33.42 ± 9.996 years. In terms of marital status, a larger portion of the participants are married (59.5%), while 40.5% are single. When examining income distribution, the data shows that a significant number of participants (36.1%) have a monthly income of over 6501 TL, with the remaining participants fairly evenly distributed among lower income brackets: 23.4% earn between 0 and 5500 TL, 18.9% earn 5501–6000 TL and 21.6% fall within the 6001–6500 TL range. Regarding internet usage, the majority of participants (36.9%) spend 1–2 h online daily, closely followed by those who spend 3–4 h online (36.4%). A smaller group reports spending less than 1 h (11.8%) or more than 5 h (14.9%) online each day. In terms of media trust and behaviour, a significant majority of participants (77.6%) do not trust online news, and a similar majority (70.0%) do verify the sources of the news they consume online (Table 1).

**Table 1:** Participant demographics and internet behaviour.

		Mean	SD
**Age**		33.42	9.996

		***N***	**%**
**Gender**	Male	427	61.6
	Female	266	38.4
**Marital Status**	Single	281	40.5
	Married	412	59.5
**Monthly Income**	0–5500 TL	162	23.4
	5501–6000 TL	131	18.9
	6001–6500 TL	150	21.6
	6501 TL and above	250	36.1
**Time Spent Online Daily**	Less than 1 h	82	11.8
	1–2 h	256	36.9
	3–4 h	252	36.4
	5 h or more	103	14.9
**Trust in Online News**	Yes	155	22.4
	No	538	77.6
**Verification of News Sources**	Yes	485	70.0
	No	208	30.0

### 2.3 Measurement Tools

Data collection was carried out online in Turkey between 01.03.2024 and 20.03.2024. The surveys were uploaded to ‘Google Forms’ by the researchers. The online survey consists of four sections. The first section includes questions reflecting personal characteristics such as gender, age, marital status and income. The second section includes the ‘Intentions to Use Violence against Health Professionals Scale’. The scale developed for the Turkish language speakers by [25] consists of the dimensions of intention to use violence, past experiences (PE), attitude towards behaviour (ATT), subjective norm (SN) and perceived behavioural control (PBC). The scale, which includes 15 statements, is structured with a 5‐point evaluation between 1: *Strongly disagree* and 5: *Strongly agree*. The Cronbach's Alpha coefficient of the scale was determined as 0.71. The third section includes the ‘Distrust in Healthcare Systems Scale’ developed by Rose et al. (2004) and adapted to Turkish language speakers by [26]. The scale consists of 10 statements and is prepared as a 5‐point Likert scale ranging from ‘1 – *I strongly disagree*’ to ‘5 – *I strongly agree*’. The Cronbach's Alpha coefficient of the scale was determined as 0.81. The last section includes the Health News Perception Scale. The scale developed for Turkish language speakers consists of 26 items and 5 sub‐dimensions (commercial concerns and advertising, orientation to consumption, negative impact on health behaviour, treatment request and abuse, and trust in health journalism) [27]. The Cronbach's Alpha coefficient of the scale was determined as 0.90.

### 2.4 Ethics

Ethics committee approval for this research was obtained from the Social and Human Sciences Ethics Committee of Dicle University, with the approval dated 04.05.2023 and numbered 488556. Participants were informed about the study through the ‘Informed Consent Form’. It was clearly communicated that participation in the study was entirely voluntary, that no names or signatures identifying personal information/identity were required, that they could withdraw from the study at any time, and that all collected data would be kept confidential. The study was conducted in full compliance with the Helsinki Declaration of Human Rights.

### 2.5 Statistical Analysis

The data analysis for this study was performed using IBM SPSS Statistics version 26. The demographic characteristics of the participants were analysed through frequency distribution, providing both the count and corresponding percentages for each category. Alongside these descriptive statistics, which included metrics such as the arithmetic mean and standard deviation, the reliability of the measurement instruments was evaluated using the Cronbach's alpha coefficient to determine the internal consistency of the scales. To explore the relationships between variables, Spearman's correlation analysis was conducted. The SPSS Process Macro was utilized to examine potential moderator effects, with Model 4 selected for this purpose. A 95% confidence interval was applied to establish statistical significance. Additionally, the robustness of the findings was reinforced through a bootstrap technique with 5000 resamples, enhancing the precision of the estimated effects and confidence intervals.

## 3 Results

As illustrated in Table 2, the analysis was conducted on 693 participants. The mean score for distrust in healthcare systems was 3.11 ± 0.64. The intention to engage in certain behaviours was notably low, with a mean of 1.32 ± 0.72. Past experiences, which also play a crucial role in shaping behaviour, had a mean score of 1.20 ± 0.65. Attitudes towards behaviour were generally negative, with a mean of 1.38 ± 0.49, while subjective norms, which gauge the perceived social pressure, had a moderate mean score of 1.83 ± 0.57. Perceived behavioural control was also moderate, with a mean of 1.77 ± 0.82. The perception of health news scored 2.94 ± 0.61. The concern regarding commercial interests and advertising in health news was higher, with a mean of 3.16 ± 0.97.

**Table 2:** Descriptive statistics for variables (*N* = 693).

Variable	*M*	SD
Distrust in healthcare systems	3.11	0.644
Intention	1.32	0.722
Past experiences	1.20	0.652
Attitude towards behaviour	1.38	0.486
Subjective norm	1.83	0.572
Perceived behavioural control	1.77	0.815
Perception of health news	2.94	0.606
Commercial concern and advertising	3.16	0.973
Consumer‐driven behaviour	2.67	1.047
Negative impact on health behaviour	2.48	0.760
Treatment desire and misuse	3.09	0.690
Trust in health journalism	3.46	0.834

Additionally, the influence of these commercial elements in driving consumer behaviour was moderate, with a mean of 2.67 ± 1.05. The perception of health news negatively impacts health behaviour had a mean of 2.48 ± 0.76. The desire for treatment and concerns about misuse, possibly influenced by media coverage, was reflected in a mean score of 3.09 ± 0.69. Lastly, trust in health journalism was relatively higher, with a mean of 3.46 ± 0.83 (Table 2).

The findings of the correlation analysis between the perception of health news and distrust in healthcare systems, as presented in Table 3, reveal several significant relationships. There is a negative and statistically significant correlation between the perception of health news and distrust in healthcare systems (*r* = −0.102, *p* < 0.01). Similarly, there is a significant negative correlation between the perceptions of commercial concern and advertising in health news and distrust in healthcare systems (*r* = −0.128, *p* < 0.01) (Table 3).

**Table 3:** Correlation analysis results for the relationship between perception of health news and distrust in healthcare systems.

	Distrust in healthcare systems
Perception of health news	−0.102*
Commercial concern and advertising	−0.128*
Consumer‐driven behaviour	0.011
Negative impact on health behaviour	0.044
Treatment desire and misuse	0.073
Trust in health journalism	0.065

* *p* < 0.01.

Other factors such as consumer‐driven behaviour (*r* = 0.011), negative impact on health behaviour (*r* = 0.044) and treatment desire and misuse (*r* = 0.073) show positive but nonsignificant correlations with distrust in healthcare systems. Lastly, trust in health journalism (*r* = 0.065) has a weak positive relationship with distrust in healthcare systems, but this correlation is also nonsignificant (Table 3).

The findings presented in Table 4 provide a detailed analysis of the correlations between various factors related to healthcare system distrust, health news perception and key behavioural variables such as intention to use violence, past experiences, attitudes towards behaviour, subjective norms and perceived behavioural control. The analysis reveals that distrust in healthcare systems is positively correlated with the intention to use violence (*r* = 0.134, *p* < 0.01). This distrust also shows significant positive correlations with past experiences (*r* = 0.083, *p* < 0.05) and attitudes towards behaviour (*r* = 0.076, *p* < 0.05) (Table 4).

**Table 4:** Correlation analysis results between intention to use violence against healthcare professionals, distrust in healthcare systems and perception of health news.

	Intention	Past experiences	Attitude towards behaviour	Subjective norm	Perceived behavioural control
Distrust in healthcare systems	0.134**	0.083*	0.076*	0.108**	0.057
Perception of health news	−0.322**	−0.275**	−0.081*	−0.126**	−0.104**
Commercial concern and advertising	−0.431**	−0.382**	−0.166**	−0.197**	−0.121**
Consumer‐driven behaviour	−0.161**	−0.130**	−0.070	−0.089*	0.037
Negative impact on health behaviour	−0.165**	−0.076*	0.066	0.063	−0.122**
Treatment desire and misuse	−0.122**	−0.174**	−0.043	−0.010	−0.057
Trust in health journalism	−0.103**	−0.081*	−0.095*	−0.028	−0.076*

** *p* < 0.05.

* *p* < 0.01.

Conversely, the perception of health news shows a significant negative correlation with the intention to use violence (*r* = −0.322, *p* < 0.01), implying that a more positive perception of health news is associated with a reduced likelihood of violent intentions. This negative relationship extends to past experiences (*r* = −0.275, *p* < 0.01), attitudes towards behaviour (*r* = −0.081, *p* < 0.05) and subjective norms (*r* = −0.126, *p* < 0.01). The analysis also highlights the role of commercial concern and advertising in shaping these relationships. There is a strong negative correlation between commercial concern and advertising and the intention to use violence (*r* = −0.431, *p* < 0.01), past experiences (*r* = −0.382, *p* < 0.01) and attitudes towards behaviuor (*r* = −0.166, *p* < 0.01). Similarly, consumer‐driven behaviour shows a significant negative correlation with intention (*r* = −0.161, *p* < 0.01), past experiences (*r* = −0.130, *p* < 0.01) and subjective norms (*r* = −0.089, *p* < 0.05). The negative impact on health behaviour shows mixed results, with a significant negative correlation with perceived behavioural control (*r* = −0.122, *p* < 0.01). Similarly, the desire for treatment and concerns about misuse are negatively correlated with intention (*r* = −0.122, *p* < 0.01) and past experiences (*r* = −0.174, *p* < 0.01). Lastly, trust in health journalism demonstrates significant negative correlations with intention (*r* = −0.103, *p* < 0.01), past experiences (*r* = −0.081, *p* < 0.05), attitudes towards behaviour (*r* = −0.095, *p* < 0.05) and perceived behavioural control (*r* = −0.076, *p* < 0.05) (Table 4).

The findings summarized in Table 5 illustrate the mediation test results, highlighting the complex interactions between health news perception, distrust in healthcare systems and the intention to use violence. The analysis indicates that the interaction between health news perception and distrust in healthcare systems has a significant effect on the intention to use violence, with a beta coefficient of 0.096 (SH = 0.036) and a 95% confidence interval ranging from 0.026 to 0.166, accompanied by a *t*‐value of −2.685. This suggests that as individuals perceive health news more positively, the adverse impact of distrust in healthcare systems on violent intentions is reduced. Moreover, the direct relationship between distrust in healthcare systems and the intention to use violence was also statistically significant, with a beta coefficient of 0.115 (SD = 0.040) and a confidence interval between 0.036 and 0.194, reflected in a *t*‐value of 2.844. Interestingly, the relationship between health news perception and the intention to use violence was highly significant as well, with a beta coefficient of −0.371 (SH = 0.043), a confidence interval from 0.287 to 0.456, and a *t*‐value of −8.654. This finding underscores the protective role of positive health news perception in mitigating the likelihood of violent intentions. Additionally, the three‐way interaction between health news perception, distrust in healthcare systems and the intention to use violence was also significant, with a beta coefficient of 0.036 (SD = 0.014) and a confidence interval from 0.008 to 0.065 (Table 5).

**Table 5:** Mediation test results.

	Bootstrap estimates		95% Confidence Interval			
	β	SD	LLCI	ULCI	*t*	*F*
Health News Perception * Distrust in Healthcare Systems	0.096	0.036	0.026	0.166	−2.685	7.210*
Intention to Use Violence * Distrust in Healthcare Systems	0.115	0.040	0.036	0.194	2.844	44.450*
Intention to Use Violence * Perception of Health News	0.371	0.043	0.287	0.456	−8.654	
Health News Perception * Distrust in Healthcare Systems * Intention to Use Violence	0.036	0.014	0.008	0.065		

* *p* < 0.01.

## 4 Discussion

The findings of this study underscore the mediating role of health news perception in the relationship between distrust in healthcare systems and intentions to use violence against healthcare professionals. These findings are particularly significant when situated within Turkey's unique cultural, political and healthcare context. Turkey's healthcare system has undergone transformative reforms over the past two decades, primarily through the Health Transformation Program initiated in 2003. While these reforms aimed to improve access and equity in healthcare, they also brought challenges, including rising costs, perceived inefficiencies and persistent inequalities in service delivery [28]. These systemic challenges have contributed to growing public dissatisfaction and distrust, positioning healthcare professionals as visible representatives of these inadequacies and making them vulnerable to aggression [29].

Media narratives in Turkey further exacerbate these dynamics. The negative perception of health news, often focused on medical errors or patient‐provider conflicts, reinforces negative stereotypes about healthcare services and professionals [30]. Such narratives contribute to a feedback loop where public frustration with the healthcare system intensifies distrust, creating an environment conducive to hostile attitudes and potentially violent behaviours. The study's findings emphasize the significant mediating role of health news perception in shaping public attitudes and highlight how negative narratives in the media exacerbate distrust in healthcare systems, aligning with recent literature on the global effects of media sensationalism [31].

Turkey's collectivist culture, characterized by strong familial and community ties, further contextualizes these findings. In collectivist societies, healthcare is often seen as a communal resource, and failures in healthcare delivery are perceived as systemic betrayals of societal trust [29]. This cultural lens magnifies public reactions to perceived shortcomings in healthcare, with frustrations often directed at frontline healthcare professionals. Additionally, Turkey's cultural emphasis on respect for authority and hierarchical structures creates high expectations of the healthcare system and its representatives. When these expectations are unmet, public disillusionment is compounded, leading to heightened distrust and, in some cases, aggression [30].

Furthermore, the comparative analysis with studies from diverse healthcare settings illustrates that similar patterns emerge globally, reinforcing the idea that negative perception of health news intensifies distrust across different national contexts. For instance, research by [32] documents the negative effects of healthcare dramatization on public trust in Scandinavian healthcare systems. Similarly, studies from other regions where the media frequently sensationalizes healthcare topics show that distrust towards healthcare providers is further exacerbated, as observed in contexts like the United States and Brazil [33, 34]. However, Turkey's unique sociocultural fabric, characterized by strong familial and communal values, influences these dynamics in ways distinct from other national contexts.

Situating these findings within Turkey's national, political and cultural context provides critical insights for non‐Turkish readers. Unlike healthcare systems in countries with higher baseline institutional trust, such as Scandinavian nations, Turkey's healthcare challenges are deeply rooted in its sociopolitical fabric. Comparisons with international contexts underscore the importance of addressing localized factors in understanding and mitigating distrust and aggression in healthcare. In global terms, this study highlights the need for targeted strategies to rebuild trust in healthcare systems, particularly in regions where systemic, cultural and media influences converge to undermine public confidence. By emphasizing the Turkish context, this research not only contributes to the existing literature but also provides a framework for addressing similar challenges in other culturally and politically complex settings.

### 4.1 Limitations

While this study offers valuable insights into the relationship between distrust in healthcare systems, health news perception and violent intentions, it is not without limitations. One of the primary limitations is the cross‐sectional design of the study, which does not allow for the establishment of causality between the variables. Longitudinal studies would be necessary to confirm the direction of these relationships over time. Another limitation is the reliance on self‐reported data, which may be subject to social desirability bias, where participants might underreport their distrust in healthcare systems or their intentions to use violence. Additionally, the study sample, although diverse, may not be fully representative of the broader population, limiting the generalizability of the findings. A second limitation of this study is the need to address the relationship between intention and actual violent behaviour. Research consistently demonstrates that while not all intentions lead to behaviour, intentions are significant predictors of potential actions. For example, studies in various contexts have shown that intention serves as a precursor to violent behaviour when accompanied by enabling conditions, such as opportunity and lack of external control mechanisms [35, 36]. In the healthcare context, intention to use violence has been linked to actual incidents of workplace violence, particularly when environmental stressors such as prolonged waiting times, perceived negligence, or sensationalized media reporting exacerbate frustrations [35, 37]. demonstrated that perceptions of imminent threat and prior exposure to violence strongly correlate with actual violent behaviours. Furthermore, while the study provides important insights into the relationship between the perception of health news, distrust in healthcare and intention to use violence against healthcare professionals, it does not account for participants' demographics, such as gender, income level and internet usage. Future research should consider these factors to provide a more comprehensive understanding.

Finally, the study is context‐specific, focusing on the healthcare system and media landscape within a particular country. As such, the findings may not be directly applicable to other settings with different healthcare systems, media practices, or cultural contexts. Comparative studies across different countries and healthcare environments would be valuable in testing the generalizability of these results.

### 4.2 Practice Implications

The findings of this study have important implications for healthcare professionals, media practitioners and policymakers. For healthcare professionals, understanding the impact of public distrust and negative media portrayals is crucial for developing strategies to improve patient relations and reduce the likelihood of violent incidents. Training programmes that focus on communication skills, empathy and conflict resolution may help healthcare workers manage potentially volatile situations more effectively. Research supports that conflict resolution training and communication skills tailored to high‐stress environments can reduce aggressive behaviours in healthcare settings [38]. Such training has proven effective in fostering safer workplace environments, particularly in healthcare, where staff regularly encounter challenging patient interactions [39].

For media practitioners, this research underscores the need for ethical health news that prioritizes accuracy and responsibility, especially in the context of reporting on healthcare systems and incidents involving healthcare professionals. The media's role in shaping public perceptions cannot be overstated, and as such, journalists and editors should be aware of the potential consequences of their reporting on public attitudes and behaviours. Balanced reporting that provides a fair representation of healthcare issues could play a pivotal role in restoring trust in healthcare systems and reducing the incidence of violence against healthcare professionals. A recent review by [40] showed that sensationalist media coverage not only impacts public perception but can lead to increased hostility towards healthcare professionals; hence, media outlets are encouraged to adopt balanced reporting practices that reinforce trust rather than erode it.

Policymakers should consider these findings when developing public healthcare strategies and communication policies. Ensuring that the public receives accurate and balanced information about healthcare systems is essential for maintaining trust and preventing violence. Policies that encourage transparency, accountability and collaboration between healthcare institutions and the media could help foster a more informed and trusting public. Additionally, public healthcare campaigns that promote positive narratives about healthcare systems and professionals could counteract the negative effects of dramatized or biased news reporting. As supported by the study by [20], establishing clear organizational policies around patient communication standards and regular staff training can serve as protective measures against violence in healthcare environments across different cultural settings.

## 5 Conclusion

This study provides a comprehensive analysis of the relationship between distrust in healthcare systems, the perception of health news and the intention to use violence against healthcare professionals. The findings highlight the significant role that health news plays in shaping public perceptions and influencing behaviour towards healthcare professionals. A key conclusion drawn from this research is that negative perceptions of healthcare systems, exacerbated by the way health news is presented, can escalate the risk of violence against healthcare professionals. The study underscores the importance of addressing these perceptions through accurate, balanced and responsible health news to mitigate such risks.

The correlation analysis revealed that a positive perception of health news significantly reduces the likelihood of violent intentions among individuals who distrust healthcare systems. This suggests that responsible health news can serve as a protective buffer, reducing the adverse effects of distrust in healthcare systems on violent behaviours. Moreover, the three‐way interaction between perception of health news, distrust in healthcare systems and intention to use violence further emphasizes the complex dynamics at play.

Finally, this study contributes to a better understanding of the complex relationships between the healthcare system, the media and public health policies by revealing the mediating role of the perception of health news in the relationship between distrust in healthcare systems and the intention to use violence against healthcare workers. The importance of conducting health news in a more ethical, balanced and responsible manner is emphasized as a means to reestablish trust in the healthcare system and prevent violence against healthcare workers.

## Author Contributions

**Selman Kızılkaya:** conceptualization, methodology, software, data curation, supervision, resources, project administration; formal analysis, writing–review and editing, visualization, validation, investigation, funding acquisition, writing–original draft.

## Ethics Statement

Ethics committee approval for this research was obtained from the Social and Human Sciences Ethics Committee of Dicle University, with the approval dated 04.05.2023 and numbered 488556.

## Conflicts of Interest

The authors declare no conflicts of interest.

## Data Availability

The data that support the findings of this study are available from the corresponding author upon reasonable request.
